# JWA regulates human esophageal squamous cell carcinoma and human esophageal cells through different mitogen-activated protein kinase signaling pathways

**DOI:** 10.3892/etm.2014.1650

**Published:** 2014-03-28

**Authors:** JIE LIN, TIELIANG MA, XIAODONG JIANG, ZHIJUN GE, WEILIANG DING, YUANYUAN WU, GUOJUN JIANG, JIAKE FENG, GUOXING CUI, YONGFEI TAN

**Affiliations:** 1 Department of Cardiac and Thoracic Surgery, Affiliated Yixing People’s Hospital, Jiangsu University, Yixing, Jiangsu 214200, P.R. China; 2 Central Laboratory, Affiliated Yixing People’s Hospital, Jiangsu University, Yixing, Jiangsu 214200, P.R. China; 3 Department of Oncology, Affiliated Yixing People’s Hospital, Jiangsu University, Yixing, Jiangsu 214200, P.R. China; 4 Department of Critical Care Medicine, Affiliated Yixing People’s Hospital, Jiangsu University, Yixing, Jiangsu 214200, P.R. China; 5 Department of Gastroenterology, Affiliated Yixing People’s Hospital, Jiangsu University, Yixing, Jiangsu 214200, P.R. China; 6 Department of General Surgery, Affiliated Yixing People’s Hospital, Jiangsu University, Yixing, Jiangsu 214200, P.R. China

**Keywords:** JWA, human esophageal cancer, small interfering RNA, mitogen-activated protein kinase signaling pathway

## Abstract

The aim of the present study was to investigate whether the JWA gene regulates the proliferation, migration and invasion of human esophageal squamous cell carcinoma (ESCC) and normal human esophageal cell lines through mitogen-activated protein kinase (MAPK) signal transduction pathways. The role of JWA in proliferation, migration, invasion and apoptosis was investigated in the Eca109 human ESCC and HET-1A normal human esophageal cell lines via transfection with JWA-small interfering (si)RNA. Western blot analysis was conducted to observe the effect of JWA on apoptosis and the regulatory effect of JWA on proliferation was determined using a thiazolyl blue tetrazolium bromide (MTT) assay. Cellular migration and invasion were analyzed via a Transwell assay. In addition, the expression levels of extracellular signal-regulated protein kinases 1 and 2 (ERK1/2), c-Jun N-terminal kinase (JNK) and p38 MAPK following JWA-siRNA transfection were detected by western blot analysis and compared with those of untreated cells. The downregulation of JWA protein decreased apoptosis and increased the proliferation, migration and invasion of the Eca109 and HET-1A cell lines. In the Eca109 cell line, the expression levels of phosphorylated (p)-ERK1/2 and p-JNK, but not those of p-p38, decreased significantly in the JWA siRNA group compared with those in the control groups. However, in the HET-1A cell line, JWA-siRNA transfection significantly inhibited the expression of p-p38 and demonstrated no effect on the expression levels of p-ERK1/2 and p-JNK. In conclusion, the JWA gene may regulate the ESCC and human esophageal cell lines through MAPK signaling pathways via different regulatory mechanisms.

## Introduction

The mitogen-activated protein kinase (MAPK) pathways are important signal transduction pathways that are key in many metabolic processes (1,2). In mammals, three predominant MAPK family members have been identified; extracellular signal-regulated kinase (ERK), c-Jun N-terminal kinase (JNK; also known as stress-activated protein kinase) and the p38 group of protein kinases (3). The ERK pathway participates in regulating cell differentiation, invasion, metastasis and opposing apoptosis. p38 MAPK has been identified to regulate microtubule polymerization and depolymerization (4). In addition, JNK and p38 predominantly regulate apoptosis, differentiation, growth and inflammatory responses (3).

JWA is a tumor suppressor gene that is commonly present in a variety of human tissues and cultured cells. The expression levels of JWA have been observed to be lower in malignant tumor tissues compared with those in non-tumor tissues (5–9). Furthermore, previous studies in mice and cervical carcinoma HeLa cells *in vitro* have shown that the expression level of JWA affects tumor proliferation, invasion and apoptosis via the MAPK pathway (9,10). However, to the best of our knowledge, the association between JWA and MAPK pathways in human esophageal cell lines has not been identified.

In the present study, the apoptosis, proliferation, migration and invasion of Eca109 human esophageal squamous cell carcinoma (ESCC) and normal human esophageal cells were observed following JWA gene knockdown. In addition, the levels of JWA protein and proteins associated with three major MAPK pathways were detected to analyze the association between MAPK, JWA and esophageal cancer.

## Materials and methods

### Materials

The Eca109 human ECSS and HET-1A human esophageal epithelial cell lines were purchased from American Type Culture Collection (Manassas, VA, USA) and cultured in RPMI-1640 culture medium containing 10% fetal bovine serum (FBS), 100 μg/ml streptomycin and 100 U/ml penicillin, in a 5% CO_2_ humidified atmosphere at 37°C. ERK1/2, JNK, p38, phosphorylated (p)-ERK1/2, p-JNK, and p-p38 monoclonal antibodies were purchased from Cell Signaling Technology, Inc. (Danvers, MA, USA). JWA, BAX and Bcl-2 antibodies were purchased from Abcam (Cambridge, MA, USA) and anti-mouse IgG (H+L) alkaline phosphatase (AP) conjugate was purchased from Promega GmbH (Mannheim, Germany). JWA-small interfering (si)RNA was purchased from Santa Cruz Biotechnology, Inc. (Santa Cruz, CA, USA).

### JWA-siRNA transfection

The cells were seeded in 6-well plates, with antibiotic-free medium containing 10% FBS for 24 h, using Lipofectamine^®^ 2000 (Invitrogen Life Technologies, Carlsbad, CA, USA) and siRNA to transfect the cells. Transfection was conducted according to the instructions provided with Lipofectamine 2000. Groups were established corresponding to 50, 100 and 150 nM concentrations of siRNA. A scrambled siRNA sequence served as the negative control (NC) group, and there was also an untreated cell group. The serum and antibiotic medium was replaced after 6 h and the total protein was extracted after 48 h. Western blot analysis was performed to identify the most effective concentration of siRNA, which was selected for subsequent experiments.

### Thiazolyl blue tetrazolium bromide (MTT; M2128; Sigma, St

Louis, MO, USA) assay. Eca109 and HET-1A cells transfected with 150 nM (the most effective concentration) JWA-siRNA were seeded in 96-well plates following 24 h of cell growth. NC and untreated groups of Eca109 and HET-1A were simultaneously seeded. After 48 h, an MTT assay was performed and the absorbance of each well was detected at a wavelength of 570 nm using a microplate reader (infinite F50; Tecan, Männedorf, Switzerland).

### Transwell assay

A Transwell assay (PIHT12R48; Millipore, Billerica, MA, USA) was performed without a Matrigel™ basement membrane to detect migration and with a Matrigel basement membrane to detect invasion of Eca109 and HET-1A cells. Twenty-four-well plates and Boyden chambers (diameter, 8 μm) were used in the present study; the cells (2×10^5^) were added to the upper chamber in a serum-free medium and the culture medium, containing 10% FBS, was added to the lower chamber. After incubating for 40 h at 37°C, the cells in the upper chamber were carefully removed and the cells from the reverse face of the membranes were harvested, fixed in methanol, stained with Giemsa and counted.

### Western blot assay

Equal amounts of protein were extracted from each group, separated by SDS-PAGE electrophoresis and transferred to nitrocellulose membranes. The membranes were blocked for 1 h with 5% bovine serum albumin in Tris-buffered saline/Tween-20 and incubated with primary antibodies overnight at 4°C. Primary antibodies included ERK1/2 (9102s), JNK (9252s), p38 (9212s), phosphorylated (p)-ERK1/2 (9101s), p-JNK (9251s), and p-p38 (9211s) monoclonal antibodies were purchased from Cell Signaling Technology, Inc. (Danvers, MA, USA) and JWA (ab173223), BAX (ab7977) and Bcl-2 (ab7973) antibodies were purchased from Abcam (Cambridge, MA, USA). Secondary antibodies [AP-conjugated anti-mouse IgG (S372B; Promega, Fitchburg, WI, USA) and AP-conjugated anti-rabbit IgG (S372B; Promega)] were added and the membranes were incubated for 2 h at room temperature. Enhanced chemiluminescence (Lumi-Phos WB; 34150; Thermo Fisher Scientific, Tewksbury, MA, USA) was used for film development, observations and radiography. Protein levels were quantified by relative to tubulin, the software used was Gel-Pro analyzer (Media Cybernetics Inc., Rockville,. MD, USA).

### Statistical analysis

Statistical significance was analyzed using SPSS software, version 14.0 (SPSS Inc., Chicago, IL, USA). The results of the quantitative analysis were expressed as mean ± standard deviation. The samples were compared using Student’s t-test or one way analysis of variance and P<0.05 was considered to indicate a statistically significant difference.

## Results

### siRNA decreases the expression of JWA

**Figure 1:**
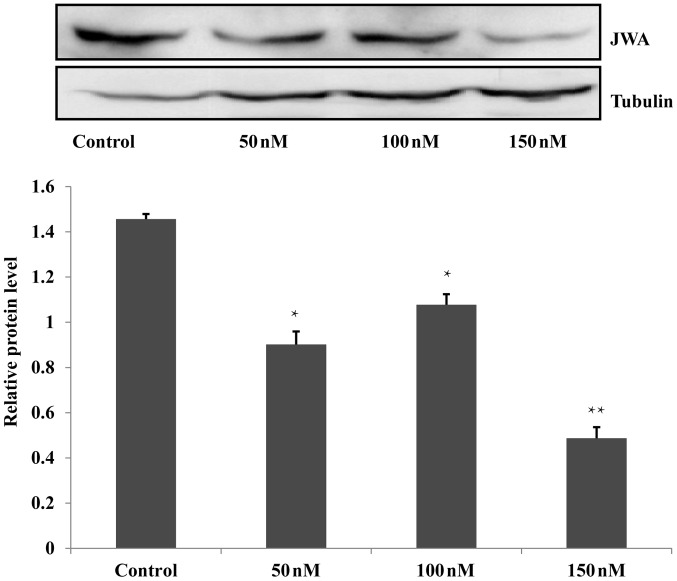
siRNA concentration test in Eca109 cells. JWA protein levels were detected following treatment with various concentrations of siRNA via western blot analysis. The 50, 100 and 150 nM concentrations of JWA-siRNA significantly decreased the expression level of JWA compared with that in the control group. ^*^P<0.05 compared with the control group; ^**^P<0.01 compared with the control group.

In the present study, RNA interference was used to interfere with the JWA gene in the Eca109 and HET-1A cells. For each cell line, the siRNA groups were treated with 50, 100 or 150 nM siRNA, a scrambled siRNA sequence was used to establish an NC group and untreated wild-type cells were included as a blank control group. Following Lipofectamine 2000-mediated transfection of the Eca109 and HET-1A cells, western blot analysis identified that 150 nM siRNA was the most effective concentration, which was selected for subsequent analysis (Fig. 1; data not shown for HET-1A cells).

### Knockdown of the JWA gene reduces cell apoptosis

**Figure 2:**
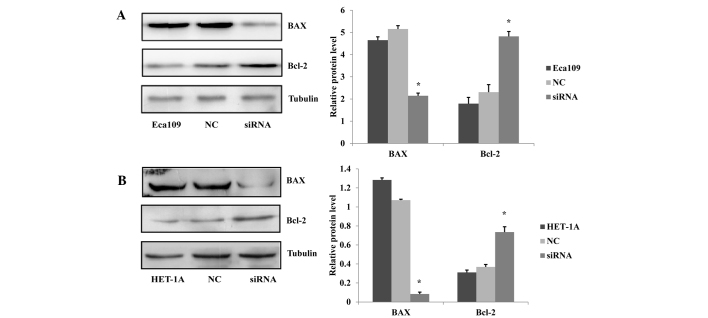
Protein expression levels of BAX and Bcl-2 detected using western blot analysis. (A) Eca109 human esophageal squamous cell carcinoma cells; (B) HET-1A human esophageal epithelial cells. In the siRNA groups, the Bcl-2 expression levels were significantly increased compared with those in the NC and untreated cells, whereas BAX expression levels decreased. ^*^P<0.05 compared with the negative control group. siRNA, small interfering RNA; NC, normal control.

The protein regulators Bcl-2 and BAX are known to independently regulate apoptosis. The Bcl-2 gene is a member of the Bcl-2 family of regulator proteins, which inhibit cell death while BAX overexpression promotes apoptosis (11). To exhibit the effect of JWA on the apoptosis of esophageal cell lines, the expression levels of the apoptosis-related proteins BAX and Bcl-2 were analyzed via western blotting. The results showed that the BAX expression levels were decreased and the Bcl-2 expression levels were increased in the siRNA-treated Eca109 cells compared with those in the NC and untreated Eca109 groups (Fig. 2A). Furthermore, in the HET-1A cells, the effect of the siRNA treatment on the BAX and Bcl-2 expression levels was similar to that observed in the Eca109 cells (Fig. 2B).

### Proliferative activity increases following JWA-siRNA transfection

**Figure 3:**
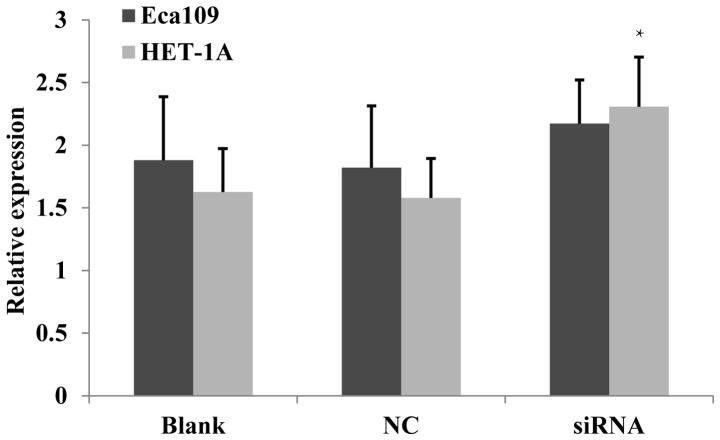
Cell proliferation activity detected by MTT assay. In the siRNA group, the proliferation activity exhibited marked enhancement compared with that of the NC and untreated (blank) cell groups. The difference was not identified to be significant for the Eca109 human esophageal cancer cells, but was significant for the HET-1A human esophageal epithelial cells. ^*^P<0.05 compared with the negative control group. NC, normal control; siRNA, small interfering RNA.

Chen *et al* (12) identified that the overexpression of JWA inhibited the proliferation of HeLa cells, whereas a low expression of JWA promoted their proliferation. In the present study, Eca109 and HET-1A cells were employed to evaluate the effect of JWA on cell proliferation following JWA-siRNA transfection. The MTT assay identified that in the Eca109 cells, the proliferation activity of the siRNA group was significantly increased compared with that in the NC and untreated cell groups (Fig. 3).

### Cell migration and invasion increase following JWA gene knockdown

**Figure 4:**
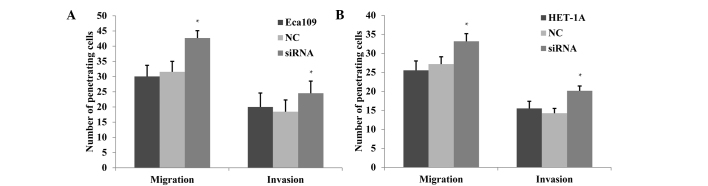
Cell migration and invasion were detected via Transwell assay. (A) Eca109 human esophageal cancer cell line; (B) HET-1A human esophageal epithelial cell line. In the siRNA groups, migration and invasion capabilities increased and the number of penetrating cells increased compared with those in the NC and untreated cell groups. ^*^P<0.05 compared with the negative control group.

Previous studies have shown a high metastatic ability for tumor cell lines when the expression level of the JWA gene is low, whereas a low metastatic ability was accompanied by a greater level of JWA expression. Bai *et al* (13) demonstrated that the migration and invasion abilities of melanoma cells were inhibited following JWA knockdown. In the present study, changes in invasion and migration capabilities following JWA-siRNA transfection were observed. The migration and invasion effects were detected via Transwell experiments, which demonstrated that in Eca109 cells, these effects were significantly enhanced in the siRNA group compared with those in the NC and untreated groups (P<0.05; Fig. 4A). In the HET-1A cells, the migration and invasion were also observed to increase significantly in the siRNA group (P<0.05; Fig. 4B).

### Protein expression of p-ERK1/2 and p-JNK in Eca109 cells and p-p38 in HET-1A cells decreases following JWA-siRNA transfection

**Figure 5:**
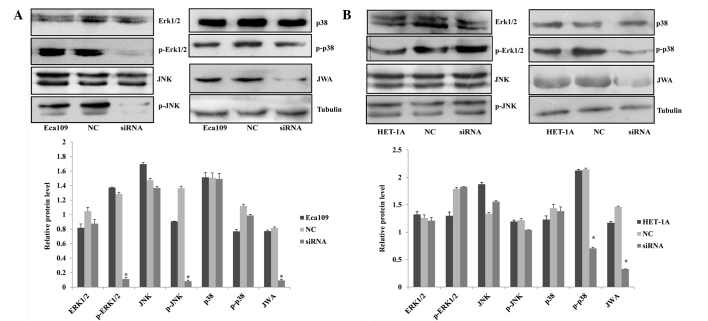
Protein expression in the mitogen-activated protein kinase pathways following RNA interference. (A) Phosphorylated (p)-ERK1/2 and p-JNK protein levels were significantly decreased in the siRNA group compared with those in the NC and untreated Eca109 human esophageal cancer cell groups (P<0.05). However, the ERK1/2 and JNK protein levels were not considered to be significantly different (P>0.05). In addition, the differences in p-p38 and p38 levels between groups were not identified as significant (P>0.05). (B) The p-p38 protein level was significantly decreased in the siRNA group compared with those in the NC and untreated HET-1A human esophageal epithelial cell groups (P<0.05), and no significant changes were observed in the levels of other proteins (P>0.05). ^*^P<0.05 compared with the negative control group. ERK, extracellular signal-regulated kinase; JNK, c-Jun N-terminal kinase; siRNA, small interfering RNA; NC, negative control.

In previous studies, JWA has been identified as a common signaling molecule of the cell signal transduction pathways induced by cancer-promoting or tumor suppressor agents; moreover, it is significant in the regulation of the MAPK pathways (14–16). Chen *et al* (12) showed that in HeLa cells, phorbol 12-myristate 13-acetate (PMA) and arsenic trioxide (AS_2_O_3_) activate MEK and ERK phosphorylation. To observe the association between JWA and the MAPK pathways, the expression of proteins associated with three MAPK pathways in the esophageal cell lines were analyzed by western blot assay. In the Eca109 cells, the protein expression level of p-ERK1/2 exhibited the most marked reduction in the siRNA group; however, the protein level of the ERK1/2 demonstrated no clear change. In another MAPK pathway, p-JNK expression was significantly decreased while the expression of JNK was not. In the third MAPK pathway, the protein levels of p38 and p-p38 did not change (Fig. 5A). However, in the HET-1A cells, the expression levels of ERK1/2, p-ERK1/2, JNK, p-JNK and p-38 did not indicate any changes. By contrast, p-p38 expression decreased following siRNA interference. These data demonstrate that the JWA regulatory mechanisms differed between the two cell lines (Fig. 5B).

## Discussion

Esophageal cancer is a predominant type of cancer worldwide. As with other malignant tumors, esophageal carcinomas are associated with changes occurring to genes. The development of esophageal cancer is a complex process involving multiple factors and stages, and numerous oncogenes and tumor suppressor genes, which alternate at the molecular level. However, the mechanism of the occurrence and development of esophageal cancer remains unclear. An insight into the mechanisms of progression and metastasis of human esophageal cell lines may provide important information for the development of therapeutic treatments.

Although the role of JWA has been investigated in various tumor cell lines (5–9), there are few studies relating to the JWA gene, which regulates proliferation, apoptosis, migration and invasion in human esophageal cells. In the present study, siRNA was used to investigate the association between JWA and MAPK signaling pathways in human esophageal cell lines.

Bcl-2 and BAX regulate cell apoptosis; overexpression of Bcl-2 inhibits cell apoptosis, which is one of the mechanisms of anticancer agents (17) and BAX overexpression promotes apoptosis to ensure that the malignant transformation of normal cells does not occur (11). Moreover, the expression of Bcl-2 and a lack of BAX generates a synergistic effect on apoptosis (13,18). In the present study, the expression of BAX was observed to decrease, whereas Bcl-2 expression increased significantly in the siRNA group. The results indicate that JWA knockdown has inhibitory effects on apoptosis in both normal and cancer cells.

Cancer cells are characterized by their rapid proliferation. In the present study, following knockdown of the tumor suppressor gene JWA, the proliferation of Eca109 and HET-1A cells accelerated. This may be associated with the cytoskeletal proteins and cell cycle characteristic of JWA (12).

JWA has been demonstrated to be a functional molecule that regulates cancer cell migration via MAPK cascades and actin filaments (12). The transfection of melanoma cells with JWA inhibited their invasive ability (13). Chen *et al* (12) indicated that the overexpression of JWA in HeLa, B16 and HCCLM3 cancer cells effectively inhibited cell migration; however, cell migration was significantly accelerated as a result of JWA knockdown. The present results indicate that migration and invasion were significantly enhanced following transfection with JWA-siRNA compared with those in untreated cells. These findings indicate that JWA inhibits the migration and invasion of normal and cancer human esophageal cells.

MAPK pathways are significant signal transduction pathways in numerous metabolic processes (1,2). Chen *et al* (12) demonstrated that in HeLa cells, PMA and As_2_O_3_ led to the phosphorylation of MEK and ERK, whereas JWA knockdown blocked MEK and ERK phosphorylation. The authors concluded that JWA regulated the cell migration via inhibition of MEK/ERK phosphorylation. Ye *et al* identified that low expression levels of JWA inhibited the RAF proto-oncogene serine/threonine-protein kinase (c-Raf)/MEK/ERK signaling pathway in MCF-7 breast cancer cells (14,19). In the present study, the levels of proteins associated with three MAPK pathways were examined in Eca109 human esophageal cancer cells; the expression level of p-ERK1/2 decreased significantly in the siRNA group, however, the protein level of ERK1/2 exhibited no clear change. These results indicate that JWA performs a regulatory role in the c-Raf/MEK/ERK signaling pathway in ESCC cells.

Zhang *et al* demonstrated that in JAr human choriocarcinoma cells, high expression levels of p-JNK and p-p38 were accompanied by increased levels of apoptosis. However, in a JWA-knockout JAr cell model, VP16 (etoposide phosphate), was unable to activate the phosphorylation of JNK and p38, thus apoptosis decreased (20). However, in the present study, a significant reduction in the level of p-JNK was observed in Eca109 cells following JWA knockdown, whereas the JNK expression did not decrease. In the third MAPK pathway, the protein levels of p38 and p-p38 did not change in the Eca109 cells; however, the p-p38 level decreased in the HET-1A cells following JWA knockdown. This revealed that there were two different regulatory mechanisms within the two cell lines.

In conclusion, it may be hypothesized that JWA regulates cell proliferation, migration and invasion via ERK1/2 and JNK pathways in human esophageal cancer cells and via p-38 in human esophageal cells. The results indicate that JWA has effects on the mechanisms that lead to the development of esophageal cancer.
