# [Expression of Concern] JWA regulates human esophageal squamous cell carcinoma and human esophageal cells through different mitogen-activated protein kinase signaling pathways

**DOI:** 10.3892/etm.2026.13289

**Published:** 2026-09-07

**Authors:** Jie Lin, Tieliang Ma, Xiaodong Jiang, Zhijun Ge, Weiliang Ding, Yuanyuan Wu, Guojun Jiang, Jiake Feng, Guoxing Cui, Yongfei Tan

Exp Ther Med 7:1767–1771, 2014; DOI: 10.3892/etm.2014.1650

Subsequently to the publication of the above article, a concerned reader drew to the authors' attention that the tubulin western blots shown in Fig. 2B on p. 1769 were strikingly similar to western blots (also reported to be tubulin blots) which appeared in Fig. 1 in the journal *Oncology Letters* in an article written by the same research group, which was published at around the same time as the above paper, although the experimental conditions for these data were reported to be different in the two articles. In addition, upon peforming an independent analysis of the data in this paper in the Editorial Office, it came to light that the JNK western blots featured in Fig. 5A on p. 1770 were very similar to the data included in Fig. 5B for the p-JNK blots, albeit the exposure times of the blots in these figure parts appeared to have been different.

The authors have been contacted by the Editorial Office to offer an explanation for these apparent anomalies in the presentation of data in their study, and we are awaiting their response. Owing to the fact that the Editorial Office has been made aware of potential issues surrounding the scientific integrity of this paper, we are issuing an Expression of Concern to notify readers of these potential problems while the Editorial Office continues to investigate this matter further.

